# Spatio-temporal field, landscape and meteorological datasets describing bruchid beetle populations, grain damage and parasitism in faba bean and lentil fields in France

**DOI:** 10.1016/j.dib.2026.112939

**Published:** 2026-06-05

**Authors:** Cayetano Herrera, Laurent Bedoussac, Anastasia Chery-Lagrange, Jean-David Chapelin-Viscardi, Yann Tricault, Antoine Gardarin

**Affiliations:** a Agronomie, AgroParisTech, INRAE, Palaiseau, France; b Univ Toulouse, Toulouse INP, ENSFEA, PURPAN, INRAE, AGIR, Castanet-Tolosan, France; c Univ Toulouse, Toulouse INP, PURPAN, INRAE, AGIR, Castanet-Tolosan, France; d Laboratoire d’Eco-Entomologie, Orléans, France; e IGEPP, INRAE, Institut Agro, Univ Rennes, Angers, France

**Keywords:** Grain legume, Pest management, Biological control, Landscape composition, Agroecology, Meteorological conditions

## Abstract

This dataset provides a multi-scale and multi-year characterization of population, grain damage and larval parasitism of bruchid beetle populations (Coleoptera: Chrysomelidae: Bruchinae) populations, i.e*. Bruchus rufimanus* Boheman, 1833 and *Bruchus signaticornis* Gyllenhal, 1833. This dataset covers 45 faba bean (*Vicia faba* Linnaeus, 1753) and 60 lentil (*Lens culinaris* Medikus, 1787) fields across four major production regions in France. Data were collected over three consecutive growing seasons (2019–2020 to 2021–2022) across three phenological crop stages (vegetative, flowering, and young pods) and at five distances from field edge to capture spatio-temporal dynamics. Datasets include bruchid counts, grain damage as well as parasitism rates by micro-hymenoptera (mainly *Triaspis* cf. *thoracica* Curtis, 1860). Field observations are integrated with environmental variables, including GIS-based landscape metrics (land use, semi-natural habitats, and hedgerow length) calculated at 500, 1000, and 2000 m buffer radii from field observation plots. Additionally, daily meteorological data from the SAFRAN platform (https://agroclim.inrae.fr/siclima/) at a resolution of 8-km grid and then aggregated per crop stage, covering temperature thresholds, precipitation, and wind speed. Agricultural management information, such as crop sowing dates, varieties, tillage and pest management, is also provided for each of the 105 fields. This integrated dataset allows for cross-disciplinary reuse in agronomy, ecology, and entomology to explore pest-enemy interactions, edge effects, and the influence of landscape and climate on crop-pest dynamics.

Specifications Table

**Table:** 

Subject	Biology
Specific subject area	Crop pest and biological regulation
Type of data	Tables (processed data): field sampling and observations, landscape characterisation and meteorological time series.
Data collection	Data were collected through field surveys conducted in 45 faba bean (*Vicia faba* Linnaeus, 1753) and 60 lentil (*Lens culinaris* Medikus, 1787) fields over three growing seasons: 2019-2020, 2020-2021, and 2021-2022. Adult bruchids were sampled using standardized protocols at different crop stages (Vegetative, Flowering, and Young pod) and at five distances from field edges. Grain samples were collected at harvest to analyse bruchid and parasitism rates. Crop development, yield components and spontaneous flora were measured *in situ*. Landscape composition was quantified using GIS-based analyses within three different buffer radii (500, 1000, and 2000 m) around each field. Meteorological data were retrieved daily from the SAFRAN platform which uses the optimal interpolation method to convert the climatic data provided by Météo-France into an 8-km grid. Data were downloaded via SICLIMA, a platform developed by AgroClim-INRAE and were aggregated over crop stages.
Data source location	Agricultural fields located in production regions in France (see locations in Fig. 1).
Data accessibility	Datasets are available in a public open-access repository (Data INRAE: 10.57745/HPEMYW): https://entrepot.recherche.data.gouv.fr/dataset.xhtml?persistentId=doi:10.57745/HPEMYW
Related research article	Chery-Lagrange, A., Gardarin, A., Tricault, Y., Gabet, M., Chapelin-Viscardi, J.-D., Herrera, C., Voisin, A.-S. & Bedoussac, L. (2026). Colonisation and spatio-temporal distribution of bruchid pests in lentil and faba bean fields. *Pest Management Science*. DOI 10.1002/ps.70679 Herrera, C., Bedoussac, L., Chery-Lagrange, A., Chapelin-Viscardi, J.-D., Tricault, Y. & Gardarin, A. Multiple spatiotemporal drivers consistently shape bruchid pest abundance, damage and parasitism in grain legumes. [under review]

## 1 Value of the Data

• These datasets provide a multi-scale and multi-year assessment of bruchid beetle populations, grain damage and parasitism in two major grain legume crops (faba bean, *Vicia faba* Linnaeus, 1753; and lentil, *Lens culinaris* Medikus, 1787) in France. The dataset includes samplings at 105 fields, at three different phenological crop stages, at five distances from field edge, and multiple environmental variables. These datasets offer a scientific basis for studying crop pest, crop damage and natural enemy dynamics across different spatial and temporal scales.
• These data can be reused to test ecological hypotheses related to crop pest colonization, edge effects and nutritional resource dynamics, landscape and meteorological drivers of infestation, grain damage and biological control. The data also enable to test the relationships between pest abundance and their damage across a range of environmental conditions. The standardized design allows for meaningful comparisons across different cropping systems or regions and supports meta-analyses as well as data re-analysis.
• The data are relevant for researchers in agronomy, ecology, and entomology, notably those working on agroecological pest management in grain legumes. Providing detailed assessment of crop pest pressure, grain damage and parasitism, these datasets can support future research, and decision-support tools in similar cropping systems.

## 2 Background

Bruchid beetles (Coleoptera: Chrysomelidae: Bruchinae) are major pests of legumes, reducing yield, nutritional value and germination potential [1,2]. Their life cycle is closely synchronized with host crop phenology and strongly influenced by meteorological conditions [[3], [4], [5]]. Adult bruchids overwinter in semi-natural habitats and colonize fields in spring while larvae develop within grains [2,6]

Literature suggests that bruchid abundance, grain damage, and parasitism may be affected at multiple spatial scales, including within-field characteristics, crop management, landscape composition, and meteorological conditions in space and time [7,8]. However, integrated datasets combining field-scale observations, landscape metrics, and meteorological variables remain limited.

The dataset was compiled to assess bruchid populations, grain damage and bruchid parasitism by micro-hymenoptera parasitoids, across multiple growing seasons and production regions in France. For this purpose, we used standardized field sampling protocols. The dataset includes GIS-based landscape analyses at several buffer radii, and meteorological data. These data support two research articles analyzing (i) colonisation and spatio-temporal distribution of bruchid pests [8] and (ii) multi-scale drivers of bruchid populations, damage and parasitism in faba bean and lentil [Herrera et al., under review].

## 3 Data Description

Data collected in 2019-2020, 2020-2021 and 2021-2022 include samplings and observations at five distances from field edge and at three crop stages in a total of 105 fields. These data allowed to assess bruchid abundance (*Bruchus rufimanus* Boheman, 1833 in faba bean and *Bruchus signaticornis* Gyllenhal, 1833 in lentil), grain damage (%) and bruchid parasitism rate (%). Dataset also includes flowering herbaceous plant cover (% cover) at field edge and within the field, number of stipules with extrafloral nectar secretion (only in faba bean), and crop- and yield-related variables. Landscape data, at 500, 1000 and 2000-m buffer around the centroid of each field studied, comprised: (i) percentages of area grown with the same crop as the focal field (faba bean or lentil) during the current and the previous sampling year, area declared as organic agriculture, grassland and woody habitats; (ii) total linear meters of hedgerows. Meteorological data, at temporal windows defined for the three crop stages, included: (i) temperature-based variables (number of days with a mean temperature above 12°C and between 20°C and 25°C); (ii) precipitation-related variables (mm) (precipitation cumulated, maximum and mean per day); (iii) maximum daily wind speed (m/s). These data were organized into eight tables (including one table for field characteristics) (Table 1), where field identification was consistent among datasets.

**Table 1:** Variables included in each dataset. Common variables such as Year, Production region, Crop (faba bean or lentil), Crop cultivated (winter faba bean, spring faba bean, or lentil), and Field are included in all datasets. Crop stage (Vegetative, Flowering, or Young pod) and Distance to field edge (m) were included only in datasets marked with an asterisk (*) and a hashtag (#), respectively.

Dataset name	Variables
Bruchids*#	Bruchid sex
	Number of bruchids individuals
Yield_and_grain_damage#	Number of bruchid-damaged grains (sum for three 0.25 m^2^ plots)
	Number of no bruchid-damaged grains (sum for three 0.25 m^2^ plots)
	Mean crop density for faba bean only (plants/m^2^)
	Yield (g/m^2^)
	Thousand-grain weight (g)
Parasitism#	Number of seeds for rearing
	Number of bruchids emerged
	Number of parasitoids emerged
	Number of seed for visual examination
	Number of bruchid holes
	Number of parasitoid holes
	Number of *Triaspis* cf. *thoracica*
	Number of Chalcidoidea
	Number of Terebrantia
	Number of Braconidae
	Number of *Anaphes* sp.
	Number of *Dinarmus* sp.
	Number of Pteromalidae
	Number of *Stethorus pusillus*
Flower_Cover*#	Area of flowering plants (%)
Extrafloral_Nectar*#	Mean number of stipules per faba bean plant with extrafloral nectar
Landscape	Area of organic agriculture (%)
	Area of crop the same year (%)
	Area of crop the previous year (%)
	Area of grassland (%)
	Area of woody habitats (%)
	Total linear meters of hedgerows (m)
Meteorological*	Number of days with temperature > 12 °C
	Number of days with temperature 20 – 25 °C
	Total precipitation (mm)
	Maximum daily precipitation (mm)
	Mean daily precipitation (mm/day)
	Maximum daily wind (m/s)
Agricultural_practice_characterization	Mode of production (organic farming, soil conservation agriculture or conventional)
	Crop variety
	Field area (ha)
	Field perimeter (m)
	Field centre (maximal surveyed distance from field edge, m)
	Intercropped species, if applicable
	Dominant vegetation at field edge (only grassy strip, hedge or woodland)
	Sowing date (dd/mm/yyyy)
	Seed origin (certified purchased seeds or farm-saved seeds)
	Seed treatment (yes/no)
	Sowing density (seeds/m^2^)
	Mouldboard ploughing (yes/no)
	Weeder harrow (number of operations)
	Rotary hoe (number of operations)
	Hoeing (number of operations)
	Pollarding (number of operations)
	Herbicides (number of treatments)
	Fungicides (number of treatments)
	Fertilisation (yes/no, specified if applicable)
	Quantity of N-fertilizer (kg N/ha)
	Yield (kg/ha)
	On-farm seed storage (yes/no)
	Presence of a legume stockpile within 2 km (yes/no)

## 4 Experimental Design, Materials and Methods

### 4.1 Study sites and experimental design

**Fig. 1:**
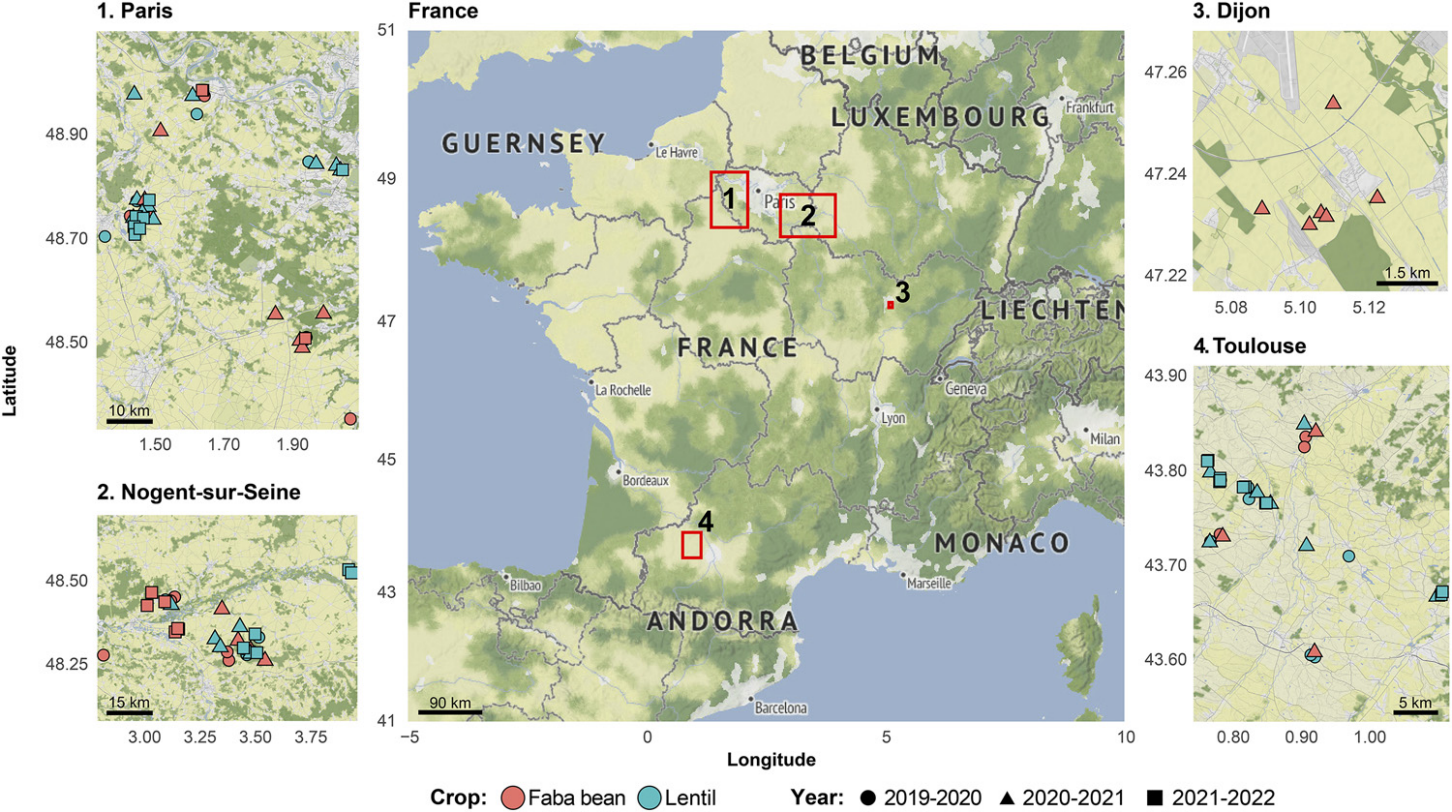
Location of the 45 faba bean and 60 lentil fields in the four production regions (near to Paris, Nogent-sur-Seine, Dijon, and Toulouse) in 2019-2020, 2020-2021, and 2021-2022.

Data were collected in 45 faba bean and 60 lentil fields, across four French production regions (near Paris, Nogent-sur-Seine, Dijon, and Toulouse, Fig. 1) during three consecutive years (2019-2020, 2020-2021 and 2021-2022). Fields were managed either organically (n=94) or under conventional practices without insecticide. Field area was on average (± SE) 8.07 ± 0.82 ha for faba bean and 8.20 ± 0.64 ha for lentil. Faba beans were sown in autumn (October 20^th^ to November 11^th^; n=35), or in spring (January 15^th^ to March 3^rd^; n=10). Lentils were sown between February 25^th^ and April 22^th^ [9].

Observations were carried out at three different phenological crop stages, i.e. vegetative, flowering, and young pods, corresponding to BBCH stages 30, 65 and 75, respectively [10] and at five distances from field edge, starting from the semi-natural habitat most represented on the perimeter of the field (grassy for 80 fields, hedge for 14 fields, and woody for 11 fields). In this field edge, we proceeded with an observation area of 10-m long and 1.50-m wide. Parallel to it and in the direction of the centre of the field, four additional observation areas of identical dimensions were defined at 5, 15, and 30 m, as well as at 50 m in 2020 and in the field centre (up to 390 m and 610 m for faba beans and lentils, respectively) in 2021 and 2022.

### 4.2 Bruchids sampling in crops

Bruchid adults within the vegetation were monitored in the field at four distances from the edge and three crop stage (105 fields x 4 distances x 3 crop stages). Insect collection was conducted using vacuum suctioning on ten randomly chosen plants in 2020 (Stihl model SH 86 C-E; air flow: 780 m³/h; air speed: 85 m/sec.) and a sweep net (Cahurel Entomologie, 50 cm diameter, 90 cm deep) in 2021 and 2022, with 20 consecutive net sweeps per transect. All insect samples were stored at -18°C immediately after collection to preserve specimens for later analysis. Sampled individuals were identified to species level and sexed using identification keys and reference material [[11], [12], [13], [14], [15]] from the Eco-Entomology Laboratory collections (https://www.laboratoireecoentomologie.com).

### 4.3 Yield and damage evaluation

Plant density (faba bean only) and grain yield were assessed at pod maturity stage in three randomly selected 0.25 m² quadrats per observation plot (105 fields x 4 distances). Grains were manually separated from the plant material, counted to determine grains per m², and dried for 48 h at 80°C. Grain were then weighted to determine the dry thousand-grain weight (g), and the yield (g/m²).

For each distance, a random sample of 25 grains was examined to record bruchid-damaged grains (grains with visible bruchid or parasitoid emergence holes or with bruchids still present) to calculate the percentage of bruchid-damaged grains.

### 4.4 Estimation of the bruchid parasitism rate

To assess bruchid biological control, we focused on larval parasitism, as oophagous parasitism was negligible [16] and predator effects could not be reliably quantified. To assess larval parasitism, 25 faba bean pods and 65 lentil pods were randomly collected at pod maturity at each observation plot (105 fields x 4 distances). Grains were manually separated from the plant material, counted, and placed into individual plastic containers sealed by fine tulle mesh and kept at 18-22°C until emergence of bruchids or parasitoids. Bruchids were identified to the species level as described above, while parasitoids were classified to the lowest feasible taxonomic level [[17], [18], [19]]. In addition, all seeds were visually inspected to record the presence of emergence holes (large holes corresponding to bruchid and small ones to parasitoids). Parasitism rate was calculated in two complementary ways: (i) reared-based parasitism rate, defined as the number of emerged parasitoids divided by the total number of emerged insects (parasitoids + bruchids); and (ii) observation-based parasitism rate, defines as the number of parasitoid emergence holes divided by the total number of emergence holes (parasitoid + bruchids).

### 4.5 Assessment of flowering plant cover and extrafloral nectar secretion

The percentage of flowering herbaceous dicotyledonous plant cover (excluding the crop species) in each quadrat was estimated visually in each crop stage, both in the field and in the field edge considering five randomly selected 1 m² quadrats along each observation plot (105 fields x 4 distances). Values were then averaged to obtain the total percentage of flowering plant cover per distance.

The number of faba bean stipules showing extrafloral nectar secretion (those with a small and shiny dot at the base) was assessed along the same plots, recording ten randomly selected plants per observation plot at each crop stage.

### 4.6 Farmer interviews and agricultural practice characterization

Field area (ha), Field perimeter (m), Field centre (maximal surveyed distance from field edge, m), and Dominant vegetation at field edge (only grassy strip, hedge or woodland) were determined using geographic information systems (GIS). To complement data, structured interviews were conducted with participating farmers to collect relevant information on their cropping practices. The questionnaire covered the following topics: Mode of production (organic farming, soil conservation agriculture or conventional), Crop variety, Intercropped species (if applicable), Sowing date (dd/mm/yyyy)¸ Seed origin (certified purchased seeds or farm-saved seeds), Seed treatment (yes/no), Sowing density (seeds/m2), Mouldboard ploughing (yes/no), Weeder harrow (number of passages), Rotary hoe (number of passages), Hoeing (number of passages), Pollarding (number of passages), Herbicides (number of treatments), Fungicides (number of treatments), Fertilisation (yes/no, specified if applicable), Quantity of N-fertilizer (kg N/ha), Yield (kg/ha), On-farm seed storage (yes/no), and Presence of a legume stockpile within 2 km (yes/no).

### 4.7 Landscape data

Spatial data were collected and analysed using GIS. Spatial layers were obtained from publicly available databases. These included the RPG (*Registre Parcellaire Graphique*) datasets for 2019, 2020, 2021, and 2022 from the National Institute of Geographic and Forest Information (https://geoservices.ign.fr/catalogue), which contain crop-specific information declared for Common Agricultural Policy subsidies, and the OSO 2023 land use database (https://theia.cnes.fr/atdistrib/rocket/#/search?collection=OSO). Data on woody habitats (e.g. woods, orchards, vineyards) were obtained from the 2024 BD TOPO layer, while hedgerow data were extracted from the 2024 French national hedgerow database (https://geoservices.ign.fr/bdhaie). The areas of semi-natural herbaceous habitats obtained from RPG data (e.g. permanent pastures) were supplemented with the OSO data for uncultivated land (e.g. lawns in green spaces). Data about organic fields (ha) were downloaded from the French government open data portal (https://www.data.gouv.fr), using the 2021 RPG Bio dataset. We assumed hedgerows and organic area remained constant over the 2020–2022 period.

Several landscape composition variables were calculated from spatial data in a buffer zone of 500, 1000 and 2000 m around the centroid of each field studied: (i) the percentage of land grown with the same crop as the focal field (lentil or faba bean), during both the current and the previous sampling year; (ii) the percentage of area declared as organic agriculture; (iii) the percentage of grassland; (iv) woody habitats; and (v) the total linear meters of hedgerows. Due to the spatial proximity of several fields, landscape data often overlapped within and across years, which may introduce non-independence in some landscape data.

### 4.8 Meteorological data

Daily meteorological data were obtained from the SAFRAN platform which uses the optimal interpolation method to convert the climatic data provided by Météo-France into an 8-km grid. Data were downloaded via SICLIMA, a platform developed by AgroClim-INRAE [20]. Each field was assigned data corresponding to its location on the 8 km x 8 km SAFRAN grid. Based on crop stages recorded during field surveys, we estimated the temporal windows corresponding to *Vegetative, Flowering*, and *Young pod* phenological stages of the crop (see workflow in Supplementary Figure 2). Within each temporal window, we calculated several meteorological variables relevant regarding bruchid activity and reproductive success: (i) number of days with a mean temperature above 12°C, considered as the threshold for activity and bruchid development [21]; (ii) number of days with a mean temperature between 20°C and 25°C, considered as an optimal temperature range for bruchid reproduction [4]; (iii) precipitation (cumulated over the temporal window, maximum per day and mean per day), which may influence oviposition or wash eggs off the pod surface [22]; and (iv) maximum daily wind speed (m/s), which can potentially interfere with mating and oviposition by the female [22].

## Limitations

Bruchid captures obtained through vacuum sampling in 2020 were very low, which led us to change the sampling methodology to sweep-net sampling during the subsequent two years. This change in sampling technique complicates the comparative analysis of data across the three sampling periods.

In addition, some samples intended for the assessment of bruchid parasitism rates were lost during transport, resulting in missing data. Furthermore, the loss of botanical survey records substantially reduces the dataset available for analysing their effect.

## Ethics Statement

This study did not conduct experiments involving humans and animals.

## CRediT Author Statement

**Cayetano Herrera:** Data curation, Writing-original draft, Visualization, Investigation, Writing-review & editing; **Laurent Bedoussac:** Funding, Conceptualization, Methodology, Writing-original draft, Visualization, Investigation, Writing-review & editing; **Anastasia Chery-Lagrange:** Conceptualization, Methodology, Investigation, Visualization; **Jean-David Chapelin-Viscardi:** Methodology, Writing-review & editing; **Yann Tricault:** Conceptualization, Methodology, Writing-original draft, Visualization, Investigation, Writing-review & editing; **Antoine Gardarin:** Conceptualization, Methodology, Writing-original draft, Visualization, Investigation, Writing-review & editing.

## Data Availability

https://entrepot.recherche.data.gouv.fr/Spatio-temporal field, landscape and meteorological datasets describing bruchid beetle populations, grain damage and parasitism in faba bean and lentil fields in France (Original data).

## Appendix Supplementary materials

Image, application 1
