# Supplementary material for: Spatio-temporal field, landscape and meteorological datasets describing bruchid beetle populations, grain damage and parasitism in faba bean and lentil fields in France
Source: Data Brief. 2026 Jun 5;67:112939. doi: 10.1016/j.dib.2026.112939 (PMC13264089; doi:10.1016/j.dib.2026.112939)
Supplement: Supplementary file 1 [file mmc1.docx]

Figure 2. Workflow illustrating the estimation of temporal windows corresponding to Vegetative, Flowering, and Young pod phenological stage of the crop.
